# Early Activation of Myeloid-Derived Suppressor Cells Participate in Sepsis-Induced Immune Suppression via PD-L1/PD-1 Axis

**DOI:** 10.3389/fimmu.2020.01299

**Published:** 2020-07-03

**Authors:** Wei-Shuyi Ruan, Meng-Xiao Feng, Jia Xu, Ying-Ge Xu, Cong-Ying Song, Li-Ying Lin, Li Li, Yuan-Qiang Lu

**Affiliations:** ^1^Department of Emergency Medicine, School of Medicine, The First Affiliated Hospital, Zhejiang University, Hangzhou, China; ^2^Department of Geriatrics, School of Medicine, The First Affiliated Hospital, Zhejiang University, Hangzhou, China; ^3^Zhejiang Provincial Key Laboratory for Diagnosis and Treatment of Aging and Physic-Chemical Injury Diseases, Hangzhou, China

**Keywords:** sepsis, MDSCs, PMN-MDSC, PD-1, PD-L1

## Abstract

**Background:** Myeloid derived suppressor cells (MDSCs) have been reported to keep elevating during sepsis. The current study was performed to investigate the immunosuppressive effect of MDSCs and their subsets with the underlying mechanisms.

**Methods:** The immunosuppressive status was manifested by the apoptosis of splenocytes, quantity of T cells and PD-1 expression. The dynamics of quantity and PD-L1 level of MDSCs and the subsets were determined over time. The subset of MDSCs with high PD-L1 level was co-cultured with T cells to observe the suppressive effect.

**Results:** Abdominal abscess was observed after 7 days post-sepsis. Five biomarkers related to organ functions were all significantly higher in the CLP group. The survival rate was consistent with the middle grade severity of sepsis model. Apoptosis of splenocytes increased over time during sepsis; CD4 + T cell decreased from day 1 post-sepsis; CD8+ T cells significantly reduced at day 7. The PD-1 expression in spleen was upregulated from an early stage of sepsis, and negatively related with the quantity of T cells. MDSCs were low at day 1 post-sepsis, but increased to a high level later; the dynamics of PMN-MDSC was similar to MDSCs. PD-L1 on MDSCs was highest at day 1 post-sepsis; PMN-MDSC was the main subset expressing PD-L1. The PMN-MDSC with high PD-L1 expression level extracted on day 1 after surgery from CLP mice significantly inhibited the proliferation of T cells.

**Conclusions:** Sepsis-induced immunosuppression is initiated from a very early stage, a high expression level of PD-L1 on MDSCs and the main subset, PMN-MDSC might play a critical role suppressive role on T cells through PD-L1/PD-1 axis.

## Introduction

Sepsis is a heterogeneous syndrome that develops as a dysregulated host response to an infection, and is associated with acute organ dysfunction which represents a high risk of death (1). Many epidemiologic studies have reported that the morbidity and mortality of sepsis are both high; thus, sepsis is still an important worldwide public health issue for a long time (2–4). Though early diagnosis, fluid resuscitation, timely delivery of antibiotics and other improvements in supportive care for critically ill patients, such as lung protective ventilation and judicious use of blood products, have distinctly improved the survival rate of sepsis at an early stage, uncontrolled primary and secondary infection resulting from sepsis-induced dysregulation of immune system may be the main cause of mortality in septic patients (5). Immunosuppression other than pro-inflammation which comes to the main status in the late stage of sepsis may be the leading cause (6).

Sepsis induced immunosuppression is characterized by lymphopenia and loss of immune function resulting from a loss of B cells and T cells via apoptosis (7). The immune checkpoint receptor, programmed cell death 1 (PD-1 or CD279), is recognized as playing a critical role in regulating the quantity and functional activity of T cells (8). Several groups have reported increased expression of PD-1 on lymphocytes after sepsis (9–11). A pilot study including 22 patients with sepsis analyzed by Wilson et al. (12) showed higher expression of PD-1 by memory subpopulations of B cells and CD4+T cells in septic patients. A subsequent study reported that sepsis enhanced expression of PD-1 on peripheral T cells and programmed cell death 1 ligand (PD-L1 or CD274) on spleen B cells and monocytes in a cecal ligation and puncture (CLP) model (13).

PD-L1, upon binding to its receptor PD-1, delivers a co-inhibitory signal to negatively regulate the activation of T-cell and mediate its apoptosis (14). Over the last decade, PD-L1 was identified in the surface of myeloid derived suppressor cells (MDSCs) in several pathological states. Noman MZ demonstrated that the expression of PD-L1 on MDSCs was significantly higher at the tumor site than at other tissues in a tumor-bearing mouse model (15). Later, Iwata T observed that the percentage of PD-L1+ MDSCs was significantly higher in hepatocellular carcinoma patients than in healthy subjects, and induced by soluble factors *in situ* such as the colony stimulating factor and vascular endothelial growth factor (16).

MDSCs are a heterogeneous group of immature myeloid cells (IMCs). Several researches have reported that MDSCs in patients with sepsis significantly elevate after onset of sepsis (17). The differentiation and maturation of IMCs are impaired during sepsis, as a result that IMCs remain as MDSCs (18), which lead to global suppression of adaptive immune function through several mechanisms, such as inducing T cell apoptosis though depleting L-arginine via iNOS, or upregulating PD-L1 (19). The major populations of MDSCs can be divided into two large groups: polymorphonuclear (PMN-MDSC) and monocytic (M-MDSC), which use different mechanisms to suppress immune responses (20). Our previous study demonstrated that MDSCs declined after hemorrhagic shock, but increased gradually after fluid resuscitation, and the ratio of M-MDSC to PMN-MDSC decreased after 24 h fluid resuscitation, but increased later (21). However, the distribution and differentiation of the subsets of MDSCs after sepsis are not well-known and few studies have demonstrated the expression of PD-L1 on MDSCs and the subsets during sepsis.

The aim of the current study is to figure out how and when MDSCs exert the suppressive role during sepsis, which subset is the main immunosuppressive group and whether PD-L1/PD-1 axis is involved in the immunosuppressive function of sepsis-induced MDSCs in a classic CLP-induced sepsis mouse model. Here, we established a CLP model of middle grade severity and hypothesized that the number and differentiation of MDSCs from different tissues might vary over time during sepsis. The main subsets of MDSC might both contribute to the suppressive effect, but one of them might be the dominant functional subset, inhibiting T cell proliferation through PD-L1/PD-1 axis. Most importantly, the suppressive progression might initiate earlier than we have previously recognized.

## Materials and Methods

### Cecal Ligation and Puncture Model

The study protocol of animal experiments was approved by the Animal Care and Use Committee of the First Affiliated Hospital, School of Medicine, Zhejiang University (Hangzhou, China). Male C57BL/6J mice aged 7–9 weeks were purchased from the Laboratory Animal Centre of Medical Institute of Zhejiang Province (Hangzhou, China). Before experiments, all of the animals were under a 12-h light and 12-h dark cycle for 1 week, in a room with controlled temperature and humidity. The mice were randomly subjected to CLP surgery or Sham operation (*n* = 5 for each group). The CLP surgery was performed as described before (22). In this study, all mice were anesthetized by intraperitoneal injection of ketamine (75 mg/kg, Sigma, San Francisco, USA) and xylazine (10 mg/kg, Sigma, San Francisco, USA). After that, the cecum was ligated 1 cm from the distal pole with a 4-0 thread. Then the ligated cecum was punctured with a 22-gauge needle midway between the ligation and the cecum tip to induce polymicrobial peritonitis. In Sham group, a similar procedure was performed on mice but without ligation and puncture of the cecum. After surgery, 1 ml of the sterile lactated Ringer's solution (Qidu, Shandong, China) was injected hypodermically to resuscitate the mice. All of the mice were given access to food and water *ad libitum*. They were closely monitored at least 3 times daily for 7 days after surgery.

### Measurement of Organ Injury Markers

Twenty-four hours after surgery, whole mouse blood was harvested. Blood was centrifuged (6,000 rpm for 15 min at room temperature) to collect serum for measurement of creatine kinase (CK) and its isoenzymes, creatine kinase-myocardial band (CK-MB), alanine aminotransferase (ALT), aspartate aminotransferase (AST), and lactate dehydrogenase (LDH). The detection kits of CK, ALT, AST, LDH were purchased from Roche (Sweden), the kit of CK-MB was purchased from SSUF Co.,Ltd (Shanghai, China). The organ injury markers were measured using an automatic biochemistry analyzer (Cobus c 701, Roche, Sweden).

### Isolation of MDSCs

Bone marrow (BM) cells were flushed from the femus and tibias of mice, spleen cells were pressed gently through a copper mesh to obtain single cell suspension. The red blood cells (RBCs) were lysed with a RBC lysis buffer (Dawenbio, Hanghou, China). The cells were then purified using a magnetically assisted MDSC isolation kit (Miltenyi Biotec, Bergisch Gladbach, Germany) with according to the manufacturer's instructions to obtain high-purity MDSCs. Briefly, the cells pellet were resuspended in running buffer (Miltenyi Biotec), FcR blocking reagent was added for 10 min at 4°C to block Fc receptor. Then, anti-Ly6G-biotin was added and incubated for 10 min at 4°C. After washed by running buffer, the cells were incubated with anti-biotin microbeads for 15 min at 4°C. Washed by running buffer again, the cells were resuspended and prepared to separation using LS column (Miltenyi Biotec) in the magnetic field of a MACS Separator (Miltenyi Biotec). After the column was rinsed with running buffer, the cell suspension was applied onto the column. The column was then washed by running buffer for 3 times, the effluent containing unlabeled Ly6G- cells were collected from the above two steps. Then the column was removed from the separator and placed on a suitable collection tube to collect the magnetically labeled Ly6G+ cells (PMN-MDSC) by pushing the plunger into the column containing 5 ml running buffer. As for isolation of M-MDSC, the steps were similar with the isolation of Ly6G+ cells. After the unlabeled Ly6G- cells were resuspended and incubated in anti-Gr-1-Biotin and streptavidin microbeads sequentially, MS column (Miltenyi Biotec) was used to collect the magnetically labeled Gr-1+Ly6G- cells.

### Flow Cytometry

Total and subsets of MDSCs were analyzed by flow cytometry. After blocking of Fc receptors, single cell suspensions from BM or spleen were incubated with the directly conjugated mouse specific monoclonal antibodies for 30 min at 4°C in the dark. After washing, about 10,000 cells were analyzed in a FACS Caliber flow cytometer using CellQuest software (Becton Dickinson, Franklin Lakes, NJ). The following antibodies were used: CD11b PE-Cy7, Ly-6C (Gr-1) PE, Ly-6G FITC, CD4 FITC, CD8a FITC and CD274 (PD-L1) APC which were purchased from eBioscience (San Diego, CA, US), and CD279 (PD-1) PE which was purchased from BD Bioscience (Sparks, MD, US).

### qPCR

Total RNA from about 5 × 10^5^ isolated PMN-MDSCs from BM of each group. The GoScript™ Reverse Transcriptase kit (Promega, Wisconsin, USA) was used to reverse transcribe RNA into complementary DNA. The 2X SG Fast qPCR Master Mix (Sangon, Shanghai, China) was used for Quantitative PCR (qPCR). The QuantStudio 3 Real-Time PCR System was used for qPCR analysis and data quantification. Used primer pairs (Sangon, Shanghai, China) derived from mouse target genes were as followed: PD-L1 forward: 5′- TGCTGCCCTTCAGATCACAG-3′, reverse: 5′- GGGCATTGACTTTCAGCGTG-3′; GAPDH forward: 5′- GACTTCAACAGCAACTCCCAC-3′, reverse: 5′- TCCACCACCCTGTTGCTGTA-3′. The expression of target gene was calculated using the ddCt method relative to the expression of GAPDH. Data shown were the relative quantity (RQ), with RQ of Sham group set to one.

### Western Blotting

Isolated Ly-6G+ cells from BM and spleen were homogenized in the lysis buffer containing 1 × RIPA buffer (Thermofisher Scientific, Waltham, MA), 1 mM PMSF (Beyotime, Shanghai, China), and 1 × Phosphatase Inhibitor Cocktail 2 (Sigma, Burlington, MA) to prepare extracts. Lysates were centrifuged at 14,000 g for 15 min at 4°C. Protein concentrations were accessed by an Enhanced BCA Protein Assay Kit (Beyotime). Equal forty micrograms of protein was loaded in 10% Tris-glycine extended polyacrylamide gels (Biorad, Hercules, CA) and then blotted on Immuno-blot PVDF membranes (Invitrogen). Membranes were blocked and incubated overnight at 4°C using the β-actin antibody (Protein Tech Group, Chicago, IL) and anti-PD-L1 antibody (ab213480, Abcam, Cambridge, UK), followed by horseradish peroxidase (HRP)-conjugated anti rabbit secondary antibody (Protein Tech Group) at room temperature for 1 h. Membranes were visualized using a Beyo Enhanced Chemiluminescence reagent kit (Beyotime, Shanghai, China) and ChemiDoc XRS + System.

### Cell Apoptosis

Spleen cell apoptosis was evaluated via the terminal deoxynucleotidyl transferase-mediated fluorescein-dUTP nick-end labeling (TUNEL) technique using the *in situ* Cell Death Detection Kit (Roche, Penzberg, Germany). The results were observed under Nikon Eclipse 50i Fluorescence Upright Microscope (Tokyo, Japan).

### Immunohistochemistry Assay

After executed with a lethal dose of ketamine and xylazine, the spleen of each mouse was taken and immersion-fixed with 10% formalin neutral buffer solution (Sangon) overnight. The spleen tissue specimens were embedded in paraffin wax after fixation and cut into 4 μm thick sections. The slides were deparaffinized in xylene and rehydrated in a graded series of ethanol (Sinopharm, Beijing, China). Then antigen retrieval was performed. Slides were incubated with proteinase K (Sigma, in 50 mM Tris Base, 1 mM EDTA, 0.5% Triton X-100, PH 8.0) for 12 min at 37°C and washed in phosphate buffered saline (PBS). Then, the slides were heated in a 96°C water bath for 20 min in the modified citrate buffer (pH 6.1, DAKO). After heating, they slowly cooled down to room temperature and were washed in PBS. Then they were treated with 0.3% H_2_O_2_ in methanol for 30 min at room temperature and subsequently incubated with blocking solution (5% goat serum) for 20 min. The primary antibody, anti-PD-1 (ZSGB-BIO, Beijing, China) was reacted with the slides overnight at 4°C. The slides were then washed with PBS three times and incubated with the HRP-conjugated anti-mouse IgG secondary antibody (Protein Tech Group) for 30 min at room temperature. The immunoreactions were visualized using a diaminobenzidine substrate kit (ZSGB-BIO). Then, all slides were counterstained with Mayer's hematoxylin (Sangon) for 20 s before washed in flowing water for 5 min. The slides were then dehydrated and immersed in xylene (Sangon) before they were cover-slipped with malinol (Muto Pure Chemicals, Tokyo, Japan). Negative controls were operated with the same procedure except the PD-1 primary antibody during incubation. For performing immunofluorescent staining, the primary antibodies were anti-Ly6G antibody (Servicebio, Wuhan, China) and anti-PD-L1 antibody (ab213480, Abcam, Cambridge, UK).

Mouse tibias were fixed in 10% formalin neutral buffer solution for 2 days, decalcified in 10% EDTA (pH = 7.4, Sinopharm) for 4 weeks and embedded in paraffin. After fixation and decalcification, the specimens were sectioned at a thickness of 4 μm for each slide for performing immunofluorescent staining. The primary antibodies were anti-Ly6G antibody (Servicebio, Wuhan, China) and anti-PD-L1 antibody (ab213480, Abcam, Cambridge, UK). All slides were observed in the P250 FLASH digital pathological system (Danjier, Jinan, China).

### Transmission Electron Microscope (TEM)

Spleen tissue samples were cut into 1 × 1 cm per piece and immediately submerged into fixation solution (Servicebio, Wuhan, China. 2.5% glutaraldehyde was the main component) overnight at 4°C. The tissue was then washed three times in the phosphate buffer (0.1M, pH7.0) for 15 min each time; post-fixed with 1% OsO4 for 1 h, it was washed three times in the phosphate butter. After double fixation, the sample was dehydrated by a graded series of ethanol (30, 50, 70, 80, 90, 95, and 100%) for 20 min at each step and then transferred to absolute acetone (Sinopharm) for 20 min. For infiltration, the specimens were submerged into 1:1 mixture of absolute acetone and final Spurr resin (Headbio, Beijing, China) mixture for 1 h at room temperature, then transferred to 1:3 mixture of absolute acetone and final resin mixture for 3 h, and to the final Spurr resin mixture for overnight. Next morning, the samples were placed in Spurr resin and heated at 70°C for 10 h and then were sectioned in LEICA EM UC7 (Leica, Germany) ultratome to 80 μm. The sections were stained by uranyl acetate and alkaline lead citrate (Sinopharm) for 5–10 min, respectively, and observed in the Hitachi model H-7650 TEM (Hitachi, Tokyo, Japan).

### Co-culture of PMN-MDSC and T Cells

PMN-MDSC suppression of T cell activation was assayed as described before (15). In this study, splenocytes from healthy mice were stained with CellTrace™ CFSE (Invitrogen™, Carlsbad, CA) at 37°C for 20 min away from light. Then same volume of complete culture medium were added and the system were incubated at 37°C for 5 min. Pelleted the cells and removed the supernatant. The stained cells were then plated into 96-well plates along with PMN-MDSCs at different ratios (5 × 10^4^ splenocytes/well, PMN-MDSC = 1/1, 1/2, 1/4, 1/8 splenocytes/well). Plates were stimulated with mouse T activator CD3/CD28 microbeads (Gibco™, Grand Island, NY) for 72 h at 37°C. The percentages of proliferating T cells were determined by flow cytometry.

### Statistical Analysis

Data analyses and graph preparations were performed using Prism 7.0 (GraphPad Software Inc.). All data values were presented as mean ± SEM. Survival curves were analyzed using a Kaplan-Meier analysis. Statistical significance of differences was evaluated by the Mann-Whitney test or unpaired two tailed Student's test. A value of *P* < 0.05 was considered statistically significant.

## Results

### CLP Model Induced Abdominal Inflammation and Organ Injury

**Figure 1:**
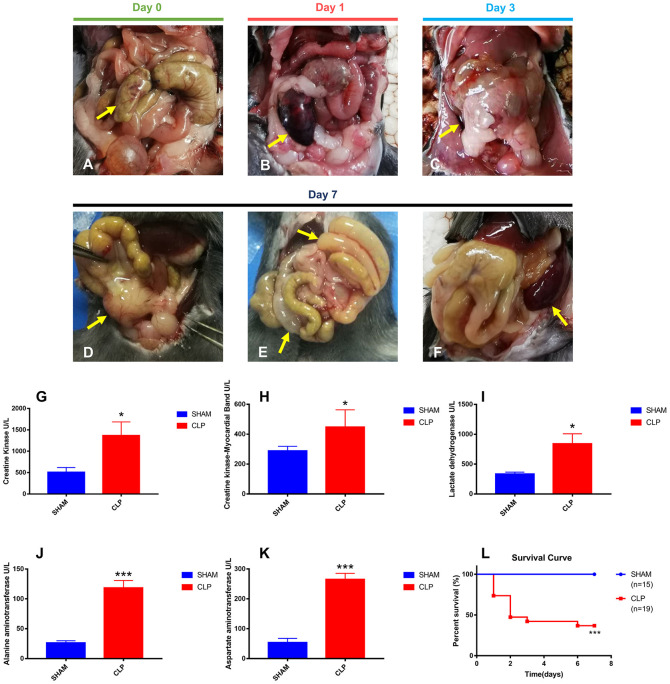
Abdominal inflammation, organ injury and survival condition after surgery. **(A)** The terminal of cecum was ligated and punctured (yellow arrow); **(B)** Avascular necrosis of ligated cecum (yellow arrow) on day 1 post-CLP; **(C)** Increased ascites and gradually adhered tissues (yellow arrow) on day 3 post-CLP; **(D)** Abscess of ligated cecum formed on day 7 post-CLP (yellow arrow); **(E)** Intestinal adhesion and obstruction on day 7 post-CLP (yellow arrow); **(F)** Splenomegaly formed on day 7 post-CLP (yellow arrow); **(G–K)** All five specific biomarkers were significantly higher in the CLP group than in Sham group (*N* = 5 for each group); **(L)** Survival rate after surgery from day 1 to day 7. For each graph, data were pooled over 3 separate time course experiments; sample sizes given are pooled over experiments. Data were expressed as mean ± SEM; ^*^P < 0.05, or ^***^P < 0.001 vs. Sham group by ANOVA.

To evaluate the severity of sepsis in the CLP model, abdominal inflammation was observed till the seventh day after surgery. The cecum was ligated and punctured in CLP surgery on day 0 (Figure 1A). The ligated cecum of CLP mice became dark red on day 1 after surgery, due to avascular necrosis caused by ligation (Figure 1B). On the third day after surgery, ascites increased obviously and the ligated cecum was gradually adhered by surrounding tissues (Figure 1C). On the seventh day, abscess formed at the site of ligated cecum, and flatulence existed due to intestinal adhesion and obstruction; moreover, the spleen became very large (Figures 1D–F). All signs of progression of abdominal inflammation after CLP surgery indicated the successfully established CLP model.

Organ injuries were assessed by specific biomarkers (CK, CK-MB, LDH, ALT and AST) 24 h after surgery. As shown in Figures 1G–K, CK and CK-MB, which were related with cardiac function, were significantly higher in the CLP group (*P* = 0.035, *P* = 0.027, respectively), indicating the injury of heart in CLP mice. ALT and AST, the famous hepatic enzymes, were also significantly higher in the CLP group (*P* < 0.001 for both markers), indicating the injury of liver in CLP mice. Increasing serum LDH levels (*P* = 0.018), can reveal the extent of tissue injury, necrosis, and hypoxia (23). In this study, the serum LDH level in the CLP group was significantly higher than that in Sham group, indicating the tissue and organ injury in CLP mice. The increased levels of biomarkers showed that at least two important organs, heart and liver, injured after surgery, which conformed to the definition of sepsis 3.0, according to the Third International Consensus Definitions for Sepsis and Septic Shock (24).

Survival condition was presented as a survival curve (Figure 1L). More than half of CLP mice died within 2 days, and only 36.84% of CLP mice survived till day 7, whereas in Sham group, all mice survived.

### T Cells Decrease Remarkably During Septic Progression

**Figure 2:**
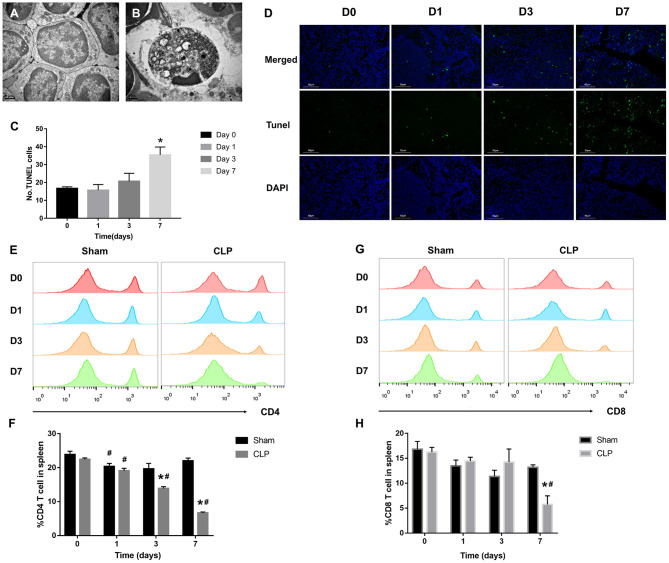
T cells decrease remarkably during septic progression. **(A)** Normal lymphocyte and **(B)** apoptotic lymphocyte were found in the spleen under TEM. Magnification: 15000×. **(C,D)** The paraffin sections were stained with TUNEL as described in Materials and Methods. Statistical analysis of the number of TUNEL-positive cells in the spleen. Three sections of each sample were prepared, and 5 random visual fields of each section were snapped. Single cell suspension of splenocytes was analyzed by FACS to determine the percentage of **(E,F)** CD4+ T cells and **(G,H)** CD8+ T cells in the spleen from Sham and CLP groups on day 0, 1, 3, 7 after surgery. Data were presented as mean ± SEM for *N* = 5 per group, ^*^P < 0.05 vs. Sham group at the same day; ^#^*P* < 0.05 vs. day 0 in the same group.

Sepsis-induced long-lasting immunoparalysis is defined, at least, by impaired T cells in the post-septic environment (25). Figure 2A showed the normal lymphocytes in the spleen of mice from Sham group; the cellular structures were well-organized and lined up tightly. However, in CLP group, more apoptotic lymphocytes appeared in the spleen (Figure 2B); chromatin gathering induced karyopyknosis; vacuole bodies appeared in the cytoplasm. To figure out whether the sepsis would induce apoptosis of splenic cells, the TUNEL assay was used on the spleen samples of mice on day 0, 1, 3, 7 after CLP. The apoptotic cells increased over the progression of sepsis, and till day 7, the positive cells increased to a significant high level than day 0 (Figures 2C,D). As we now know, apoptosis increased in spleen, and then what happened to the T cells inside it? With FACS analysis, the quantity of CD4+ and CD8+ T cells were easily determined. Figures 2E,F showed that the percentage of CD4+ T cells decreased not only in the late stage of sepsis, but also from the beginning. CD4+ T cells of spleen in the CLP group decreased significantly from day 1, and continually declined to a very low level in the late stage. However, CD4+ T cells in Sham group decreased slightly on day 1, and rose back to a normal level later. The variation of CD8+ T cells in spleen was similar with CD4+ T cells, though CD8+ T cells did not reduce significantly before day 3, it declined to a low level on day 7 either (Figures 2G,H). The results demonstrated that T cells exhausted in the late stage of sepsis, but apoptosis began very early especially for CD4+ T cells.

### PD-1 Is Upregulated on T Cells in the Spleen During Septic Progression

**Figure 3:**
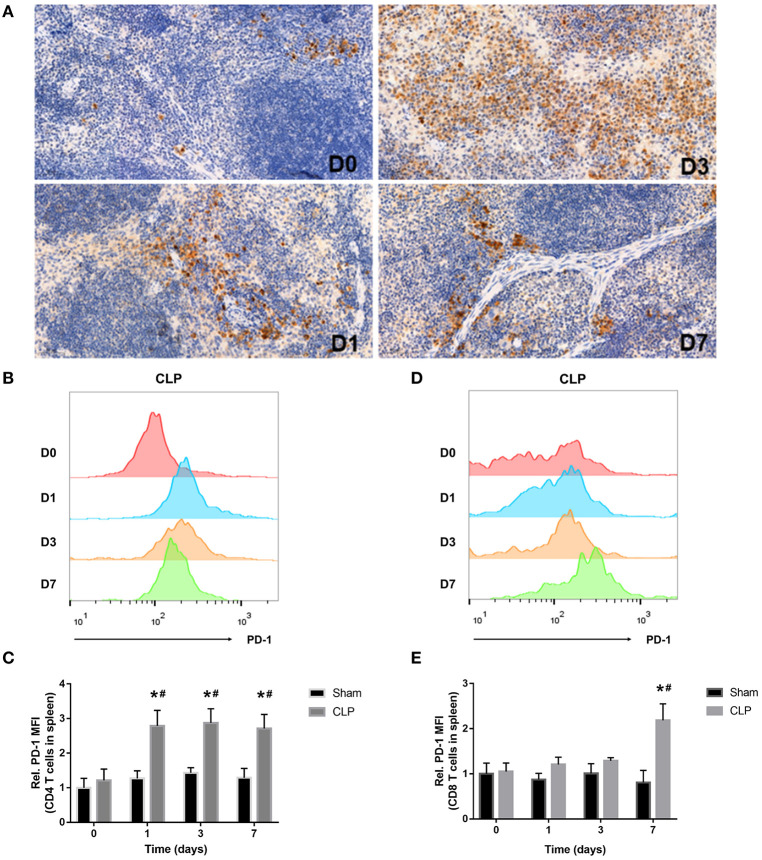
PD-1 is upregulated on T cells in spleen during septic progression. **(A)** Expression of PD-1 in the spleen on day 0, 1, 3, 7 after CLP. Paraffin sections of spleen were stained with the monoclonal anti-PD-1 protein antibody as described in Materials and Methods. The images were captured at magnifications under 200× objectives. **(B,C)** Surface expression of PD-1 on CD4+ and **(D,E)** CD8+ on day 0, 1, 3, 7 after surgery. The data were presented as mean ± SEM for *N* = 5 per group. ^*^P < 0.05 vs. Sham group at the same day; ^#^*P* < 0.05 vs. day 0 in the same group.

The inhibitory receptor PD-1, a negative regulator of activated T cells, is reported to be upregulated in sepsis patients (12, 26). In our research, immunohistochemistry and flow cytometry were applied to detect the expression of PD-1 in the spleen and on T cells. Immunohistochemistry staining of the splenic sections on day 0, 1, 3, 7 after CLP showed that PD-1 was at a low grade on day 0, whereas, from day 1, PD-1 was upregulated evidently and the positive cells increased. On day 3, the expression of PD-1 was inclined to a high level and the positive cells increased remarkably, but till day 7, though PD-1 was still at a high level, it seemed that positive cells reduced and were less than day 3 (Figure 3A). When targeting to T cells, FACS analysis showed that surface expression of PD-1 on CD4+ T cells was upregulated from day 1 and keeping at a high level during sepsis. But surface expression of PD-1 on CD8+ T cells was upregulated until day 7 (Figures 3B–E). The FACS results were corresponding to the count of each T cell that when PD-1 was upregulated, the number of T cells decreased.

### MDSCs Decrease in the Early Stage and Increase in the Late Stage

MDSCs may involve in immunosuppression during sepsis, and the quantity of MDSCs was an important parameter. The variation in quantity of MDSCs and the subsets was evaluated by the cells from bone marrow and spleen using flow cytometry. MDSCs were labeled as CD11b+ Gr-1+ cells. The two main subsets of MDSCs, PMN-MDSCs and M-MDSCs, were labeled as CD11b+Ly6G+Ly6C^low^ and CD11b+Ly6G-Ly6C^high^, respectively (27). In addition, we also used TEM to detect the cellular morphology in spleen, where the MDSCs might interact with T cells.

**Figure 4:**
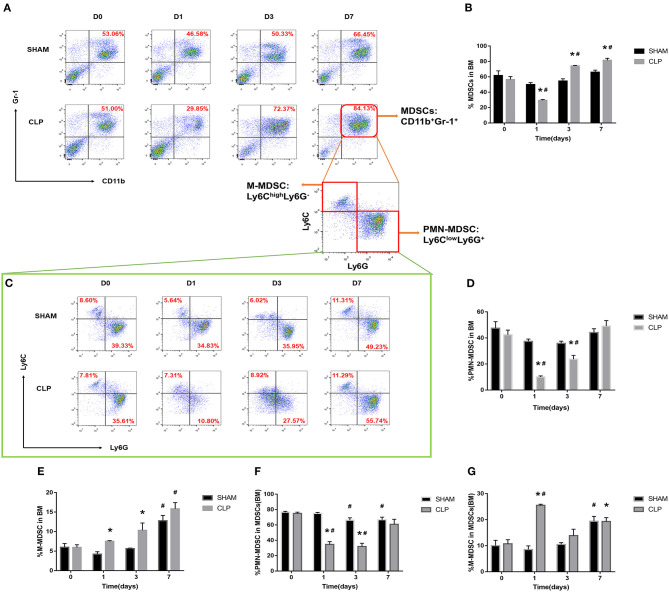
The quantity of MDSCs and the subsets in bone marrow. BM cells were isolated from Sham and CLP mice, and CD11b+ Gr-1+ MDSCs were quantified by flow cytometry. **(A,B)** Representative flow cytometry plots and the statistical graph showed the percentage of CD11b+ Gr-1+ MDSCs in BM after 0, 1, 3, 7 day post-surgery. **(C–G)** The representative plots and relevant statistical graph showed the percentage of CD11b+ Ly6C^low^Ly6G+ PMN-MDSC and CD11b+ Ly6C^high^Ly6G- M-MDSC in BM and in total MDSCs derived from BM. Data were shown as mean ± SEM from *N* = 5 per group, ^*^P < 0.05, vs. Sham group at the same day; ^#^*P* < 0.05, vs. day 1 in the same group.

The MDSCs and the subsets from bone marrow were detected at the time of day 0, and 1, 3, 7 after surgery. The data and graph in Figures 4A–G indicated that MDSCs from BM decreased significantly on day 1 after CLP. However, on day 3, MDSCs increased significantly to a high level, which was more than that in Sham group and day 1 of CLP group. On day 7, MDSCs continued to increase to a higher level. PMN-MDSCs, the main subset, had the similar variation trend as MDSCs. However, M-MDSCs, only a small part of MDSCs, kept elevating after CLP. The percentages of the subsets in MDSCs were roughly similar to those in BM cells, but the percentage of M-MDSC in MDSCs derived from bone marrow dramatically increased on day 1, which was mainly because that both MDSCs and PMN-MDSC decreased vigorously.

As for spleen, the function, which is in combination with a highly organized lymphoid compartment, makes it the most important organ for antibacterial and antifungal immune reactivity (28). Thus, the spleen plays an important role in the immune regulating process of sepsis. MDSCs, the symbol of immunosuppression, may inhibit proliferation of lymphocytes or induce cellular dysfunction.

**Figure 5:**
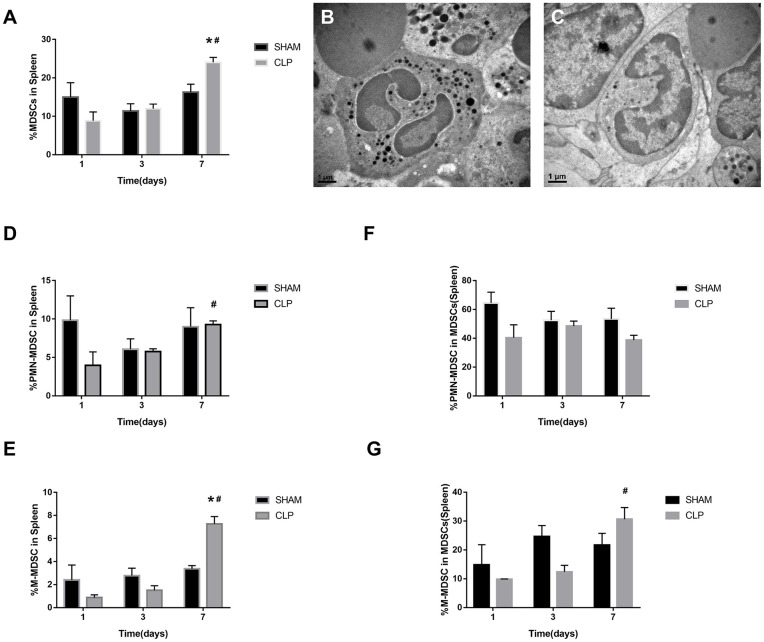
The presence of MDSCs and the subsets in spleen. **(A)** The graph showed the percentage of CD11b+ Gr-1+ MDSCs in spleen after 1, 3, 7 day post-surgery. **(B)** Polymorphonuclear cells and **(C)** monocytic cells were found in spleen under TEM. Magnification: 15,000×. **(D–G)** The percentage of CD11b+ Ly6C^low^Ly6G+ PMN-MDSC and CD11b+ Ly6C^high^Ly6G- M-MDSC in splenocytes and in total MDSCs derived from spleen. Data were shown as mean ± SEM from *N* = 5 per group, ^*^P < 0.05, vs. Sham group at the same day; ^#^*P* < 0.05, vs. day 1 in the same group.

The data in Figures 5A,D–G indicated that MDSCs and the subsets decreased on day 1 after CLP, but increased continually during the septic progression, and to a significantly higher level till day 7. The trend was generally similar to the results of bone marrow, but in spleen, there was no significant increase of MDSCs at day 3. Mobilization of BM to the peripheral organs may lead to such delay in spleen. M-MDSC increased continually in spleen. However, the percentage of PMN-MDSCs in MDSCs derived from spleen had no significantly different change during sepsis. In the meantime, polymorphonuclear cells and monocytic cells were found in spleen (Figures 5B,C) under TEM.

### PD-L1 Is Upregulated on MDSCs Especially on PMN-MDSC in Septic Mice

**Figure 6:**
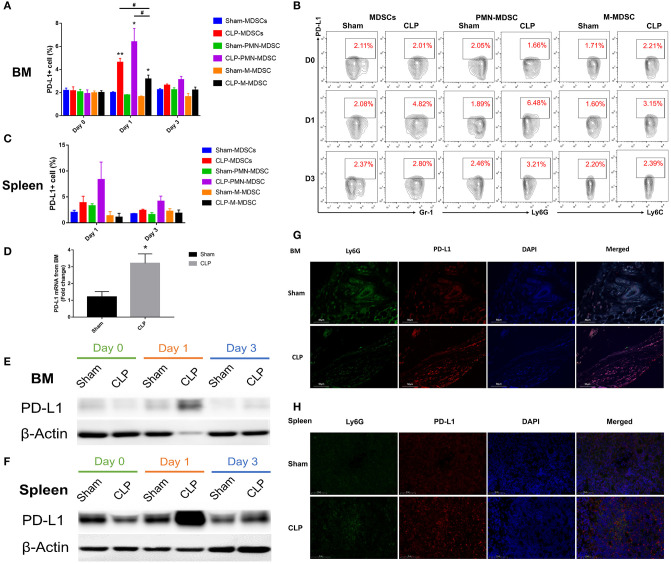
PD-L1 is upregulated on MDSCs especially PMN-MDSCs in septic mice. **(A)** Surface expression of PD-L1 on MDSCs and the subsets from BM on day 0, 1, 3 after surgery. **(B)** Representative flow cytometry graphs of Surface expression of PD-L1 on MDSCs and the subsets from BM. **(C)** Surface expression of PD-L1 on MDSCs and the subsets from spleen on day 1, 3 after surgery. **(D)** mRNA level of PD-L1 on MDSCs and the subsets from BM on day 1. **(E,F)** Protein expression of PD-L1 from isolated PMN-MDSC derived from BM and spleen on day 0, 1, 3 post-surgery analyzed by western blotting. **(G,H)** Immunofluorescent double staining Ly6G and PD-L1 of BM and spleen from mice of each group. Data were presented as mean ± SEM. *N* = 5 per group, ^*^P < 0.05, ^**^P < 0.01, vs. Sham group on the same day; ^#^*P* < 0.05, M-MDSC vs. MDSCs or PMN-MDSC.

PD-L1 is a ligand of PD-1, and it is expressed on tumor cells and immune cells. PD-L1+ cells may interact with T cells and induce PD-1 overexpression on T cells, leading to T cell apoptosis and immunosuppression. In order to determine whether the co-inhibitory factor PD-L1 was involved in the immune function of MDSC, flow cytometry was used to access the PD-L1 expression on surface of MDSCs and the subsets from bone marrow of each group. Figures 6A,B showed that the surface expression of PD-L1 on MDSCs from BM was upregulated to a significantly higher level than Sham group on day 1 after CLP, while on day 3, the expression diminished. The data indicated that the peak of PD-L1 expression on the surface of MDSCs appeared on day 1. Both PMN-MDSC and M-MDSC had more percentage of PD-L1+ cells in CLP group than those in Sham group. Furthermore, in CLP group, PMN-MDSC had more percentage of PD-L1+ cells than M-MDSC on day 1, but had no significantly difference with MDSCs. This result suggests that PMN-MDSC is likely to be the dominant subset expression of PD-L1. Thus, we investigated the mRNA level of PD-L1 on PMN-MDSC from BM especially on day 1. The result showed that mRNA level of PD-L1 in CLP group was also significantly higher than that in Sham group (Figure 6D). In addition to BM, we measured the expression of PD-L1 on MDSCs from spleen, where the cells exerted immune regulating function. The surface expression of PD-L1 on MDSCs from spleen had similar tendency with the cells from BM, though the statistical differences were not significant (Figure 6C). The protein level of PD-L1 in PMN-MDSC from both BM and spleen of CLP mice were remarkably higher on day 1 than those of Sham group (Figures 6E,F). The immunofluorescent double staining shown in Figure 6G presented that the Ly6G+ cells (the specific marker of PMN-MDSC) from BM were remarkably less in CLP group than that in Sham group on day 1 after surgery, whereas PD-L1 level was obviously higher in the CLP group at the same time. This experiment well-combined the quantity of PMN-MDSC with the expression of PD-L1, to illustrate the overall increased expression of PD-L1 on day 1 despite the decreased number of PMN-MDSC. In spleen, the expression of PD-L1 were obviously higher on Ly6G+ cells in CLP group on day 1 either, whereas Ly6G+ cells were not decreased (Figure 6H). The results above strongly demonstrated that PD-L1 on MDSCs, especially on PMN-MDSCs, was upregulated at a very early stage after sepsis.

### The Increase in the Number of T Cells Is Inhibited by PMN-MDSC From CLP Mice

**Figure 7:**
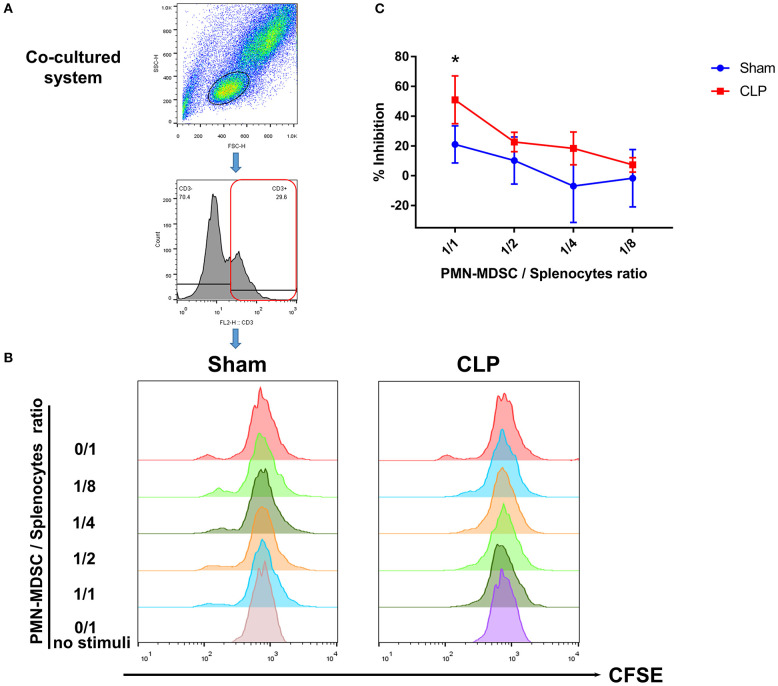
T cell proliferation is inhibited by PMN-MDSC from CLP mice. **(A)** Gating strategy of CFSE+ T cells. **(B)** CD11b+ Ly6C^low^ Ly6G+ PMN-MDSC isolated by microbeads assisted cell sorting was co-cultured with healthy CD3+ T cells stimulated by anti-CD3/CD28 microbeads at the ratio of 0/1,1/1,1/2,1/4,1/8 (PMN-MDSC/T). Representative histograms showed the proliferation of activated T cells in the presence of PMN-MDSC from Sham and CLP mice. No stimuli means T cells without stimulated by mouse T-activator CD3/CD28 microbeads. **(C)** Statistical analysis of the inhibition of proliferated T cells. Data were shown as mean ± SEM from *N* = 5 per group. ^*^P < 0.05.

The results above suggested that PMN-MDSC might be the main subset exerting the suppressive effect via PD-L1. Thus, to verify whether post-septic PMN-MDSC possessed suppressive activity, BM PMN-MDSC from both group on day 1 were isolated, and co-cultured with healthy mice-derived splenocytes in which T cells were activated by anti-CD3/CD28 microbeads. We observed that though PMN-MDSC from Sham mice had some ability to inhibit the proliferation of T cells when the proportion of PMN-MDSC was high, PMN-MDSC derived from CLP mice gained a stronger ability of inhibition in a ratio dependent manner, the difference was significant between the two groups when the cell ratio was 1/1 (Figures 7A–C). Collectively, we demonstrated that PMN-MDSC which expressed high level of PD-L1 after CLP exhibited immunosuppressive properties that limited the increase in the number of T cells in septic mice.

## Discussion

The present study demonstrated that PD-1 was upregulated on spleen CD4+ T cells and CD8+ T cells during sepsis, which represented the immunosuppressive state of CLP mice. The quantity of MDSCs was low at the early stage but later continually increased to a higher level than normal in both BM and spleen of CLP mice. PD-L1 was upregulated on MDSCs especially on the main subset, PMN-MDSC, from CLP mice at an early stage of sepsis, which could enhance the suppressive effect of the cells. Finally, the increasing apoptosis and inhibited proliferation of T cell reminded that sepsis-induced MDSCs with high PD-L1 expression, especially PMN-MDSC, may exert immunosuppressive role by inducing T cell exhausting through PD-L1/PD-1 axis from a very early stage of sepsis.

Polymicrobial sepsis induced by CLP surgery is a common model because it closely mimics the progression and characteristics of human sepsis (29). According to the study conducted by Daniel, we ligated around the medium of cecum, and punctured using a 22G needle to establish a mid-grade sepsis model; the survival rate in this study was 36.84% after 7 day of sepsis, consistent with the previous study where the survival rate of mid-grade sepsis mouse group was around 40% (22). Though the CLP model was regarded as a golden standard model for sepsis, little study has testified the model according to new sepsis definition (sepsis-3) from 2016, where the sepsis was redefined as life-threatening organ dysfunction caused by a dysregulated host response to infection (24). Here, we demonstrated that at least two organs (such as heart and liver) were impaired after CLP, and proved that the model conformed to the new sepsis definition. In addition, the spleen which was an important immune organ of CLP mouse, remarkably enlarged over time, where the physiological activation of immune cells should be paid great attention to.

The spleen plays an important role in responding to infections. A higher percentage of immune cell depletion was detected in the spleen of septic patients compared to other organs (30). Specific subsets of lymphocytes may be more vulnerable under such conditions. Zhou demonstrated in a research that the splenic CD4+T cells reduced over 3 days after CLP (31). In the present study, the apoptotic cells increased in spleen over time, and were significantly higher at day 7 (late stage of sepsis) than those of day 0 post-CLP. The two main subsets of T cells, CD4+, and CD8+ T cells, both reduced after CLP surgery, and CD4+ T cells decreased from an early stage while CD8+ did not significantly reduce until day 7. The apoptosis of splenocytes and specific T cells enhanced the dysregulation of immune system of CLP mice.

The co-inhibitory receptor PD-1 maintains immune homeostasis by negatively regulating T cell survival (32). Brahmamdam once reported that the anti-PD-1 antibody could prevent sepsis-induced depletion of lymphocytes and improve survival (33). Therefore, we detected the expression of PD-1 in spleen and observed that PD-1 in spleen tissue of CLP mice increased *in situ* during sepsis. Then specific subsets of T cells were targeted, and the expression levels of PD-1 on CD4+ and CD8+ T cells were upregulated but negatively correlated with their quantities. These results were consistent with the previous studies (34). Comprehensively, PD-1 increased from a very early stage on spleen T cells particularly on CD4+ T cells, which induced the apoptosis of T cells. In the late stage, the effect accumulated and contributed to sepsis-induced immunosuppression.

PD-L1 is a well-known ligand of PD-1. Blockade of PD-L1/PD-1 axis has been used in several disorders, such as cancer and infection (35–37). PD-L1 is expressed extensively on immune cells, including B cells, macrophages, DCs, BM-derived mast cells etc (38). Von Knethen observed that PD-L1 was upregulated in the liver early after 1 day post-sepsis (39). MDSCs are pathologically activated and specialized immunosuppressors which can control the functions of other immune cells including T cells (40). The suppressive activity of MDSCs is mediated by nitric oxide, S100A8 and S100A9 proteins, etc (41). Recently, PD-L1 was reported as a novel marker on MDSCs to mediate the immunosuppressive function (42). In the present study, the expression level of PD-L1 and its immunological role on MDSCs and the subsets were investigated.

The myelopoiesis-biased process triggered by inflammation increases the generation of not only mature myeloid cells, but also immature myeloid progenitors and MDSCs. MDSCs will be recruited into inflamed tissues where they suppress acute inflammatory responses; however, long-term presence of MDSCs suppresses the host's immune system, and increases susceptibility to infection (43). Hollen observed that MDSCs kept elevating in sepsis survivors for at least 6 weeks after infection, but only MDSCs obtained 14 days and later post-sepsis had a significant suppressive function (44). In the current study, we counted the number of MDSCs in BM and spleen, and observed that the number of MDSCs from BM was small on day 1 post-CLP while increased significantly to a high level on day 3, which is considered entering the late stage of sepsis. Though the percentage of MDSCs was much less in spleen than in BM, and early decline was not remarkable, the amount of dynamics was similar. The results were essentially consistent with the outcomes observed by Brudeki that the quantity of MDSCs was small at the beginning of sepsis, while increased over 75% of BM cells 6 days later (45). What's more, our study counted the number of the two major subsets of MDSCs: PMN-MDSC, the dominant subset, had the similar variation trend as MDSCs, in the meantime, M-MDSC kept elevating after CLP. Several studies have demonstrated that these two subsets owned their different mechanisms in sepsis, mostly because PMN-MDSC was more like neutrophil and M-MDSC was close to monocyte (46–48). The slight difference was that PMN-MDSCs in our study were higher on day 0 than on day 1. We made an assumption that a group of cells sharing the same phenotype as PMN-MDSC, but with proinflammatory role, such as neutrophils, might be mobilized from BM to other tissues on day 1 post-sepsis, so the quantity of PMN-MDSC in BM seemed lower than day 0.

Lu C observed in their study that PD-L1+ MDSCs were higher in tumor situ, and BM MDSCs were essentially PD-L1- in tumor bearing mice (49). But unlike tumor, a lipopolysaccharide (LPS) induced sepsis mice model established by Landoni showed that BM MDSCs already had the potential to inhibit T-cell proliferation (50). We observed in this research that, though the number of BM MDSCs on day 1 post-CLP was lower than on day 0, the surface expression of PD-L1 on BM MDSCs was upregulated on the emerged cells at an early stage of sepsis, which may be associated with the activated suppressive role of BM MDSCs. However, the expression of PD-L1 returned to a low level at day 3 while the number of MDSCs increased. The phenomenon suggested a possibility that the underlying mechanisms of immunosuppressive function of MDSCs induced by sepsis might be changing over time. The suppressive role of MDSCs onset at the early stage of sepsis might be played through upregulation of PD-L1, while the increased MDSCs later might be activated in other ways.

PMN-MDSCs, the most abundant population of MDSCs in both mouse and human, share many morphological and phenotypic characteristics of neutrophils, but are immunosuppressive (51). Recently, Tsukamoto demonstrated that M-MDSC from spleen exhibited higher expression of PD-L1 comparing with PMN-MDSC after sepsis (52). However, in another study, Fu discovered that MDSCs, particularly PMN-MDSCs, increased after emergency myelopoiesis of pulmonary hypertension, and PD-L1 expression was elevated on circulating PMN-MDSC from patients with pulmonary (53). Emergency myelopoiesis including granulopoiesis represents a physiological response of the immune system to infection after sepsis (54). Thus, we not only detected the number of the subsets, but also investigated the expression of PD-L1 on the two subsets of MDSCs. Finally, though PD-L1+ cells increased on both subsets from BM, the positive percentage was higher on PMN-MDSC than on M-MDSC. The result was different from what Tsukamoto found in spleen. Hence, we then focused on the expression and function of PMN-MDSC. The mRNA, surface and protein level of PD-L1 on BM PMN-MDSC were all relatively higher after 1 day post-CLP than post-Sham. The results suggested that the onset BM PMN-MDSC with high PD-L1 might already have the suppressive role. Though the total volume of protein was same for each group in western blot experiment of BM PMN-MDSC, the results showed that the protein level of housekeeping gene, β-actin, was especially lower in CLP group on day 1, whereas PD-L1 protein was significant overexpressed in the same group within every replication. The phenomenon suggested that in the acute stage of sepsis, when housekeeping proteins could be affected as well, the control for variations in protein loading might require new technologies, e.g., total protein stain.

In addition to BM, the significant overexpression of PD-L1 on PMN-MDSC from spleen on day 1 post-CLP further strongly supported that the suppression role of PMN-MDSC through PD-L1/PD-1 occurred from very early after sepsis. Thus, in a following experiment, PMN-MDSC on day 1 post-CLP were extracted and co-cultured with T cells. Though we observed that the proportion of responding T cells was low when only stimulated by mouse T-activator CD3/CD8 microbeads, the results still verified the hypothesis that BM PMN-MDSC with high PD-L1 from day 1 of CLP mice had a significant immunosuppressive role of T cell proliferation than those with low PD-L1 from Sham mice. The result indicated that the suppressive activity of MDSCs, particularly PMN-MDSC, was mediated by PD-L1/PD-1 axis at an early stage of sepsis.

Our study has not only revealed the PD-1 expression in spleen, but also demonstrated the timing of PD-L1 emerging in MDSCs after sepsis. Thus, a precise immune modulation therapy administered earlier more than current treatment opinions to maintain low count of MDSCs or block PD-L1/PD-1 axis after sepsis may be promising for better prognosis.

There are several limitations in this study. First, we did not clarify why initial percentage of MDSCs was higher than that of day 1. According to our assumption, further studies are required to investigate the role of cells sharing similar phenotype with MDSCs and separate the real immunosuppressive phenotype of MDSCs, PD-L1 may be the critical biomarker. Second, the molecular mechanisms regulating the PD-L1 expression and the immunosuppressive mechanism of MDSCs in the late stage of sepsis were not demonstrated in this study; the increased MDSCs must be further investigated to elucidate the mediators of suppressive activity over the whole progression of sepsis. Finally, the immunosuppressive function of M-MDSC was not manifested in this study, because the amount of M-MDSC was so small that made it difficult to extract.

## Conclusions

**Figure 8:**
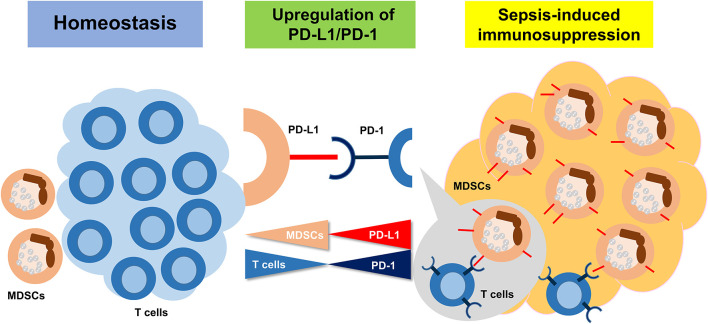
The summary of the study. Sepsis may induce an immunosuppression state, resulting in MDSC expansion. Upregulation of PD-L1 on MDSCs is ligated to increasing PD-1 on T cells and inducing the T cell apoptosis.

In conclusion, sepsis-induced immunosuppression is initiated from a very early stage, represented by apoptosis of splenocytes, decreased CD4+ and CD8+ T cells, and a high expression level of PD-L1 on MDSCs. PMN-MDSCs, the dominant subset of MDSCs, play an important suppressive role through PD-L1/PD-1 axis at the early stage of sepsis (Figure 8).

## Data Availability Statement

The raw data supporting the conclusions of this article will be made available by the authors, without undue reservation, to any qualified researcher.

## Ethics Statement

The animal study was reviewed and approved by the Animal Care and Use Committee of the First Affiliated Hospital, School of Medicine, Zhejiang University (Reference Number: 2019694).

## Author Contributions

W-SR contributed to the design, observed abdominal inflammation and survival using CLP models, prepared tissue samples, analyzed the surface phenotypes of immune cells by FACS, and performed the co-culture experiments. W-SR and M-XF prepared the bone marrow MDSCs and splenocyte single suspension, performed magnetically assisted cell sorting, and the FACS staining. JX studied the tissue after staining with PD-1 and provided guidance for T cell studies. Y-GX and C-YS analyzed the protein expression using western blotting and studied the tissues after staining with Ly6G and PD-L1. L-YL studied tissues using Transmission electron microscope. LL assisted CLP surfery and co-culture experiments. Y-QL supervised the study, analyzed the data, and wrote the manuscript together with W-SR. All authors contributed to the article and approved the submitted version.

## Conflict of Interest

The authors declare that the research was conducted in the absence of any commercial or financial relationships that could be construed as a potential conflict of interest.
